# Prospective clinical study of rehabilitation interventions with multisensory interactive training in patients with cerebral infarction: study protocol for a randomised controlled trial

**DOI:** 10.1186/s13063-017-1874-y

**Published:** 2017-04-11

**Authors:** Wai Leung Ambrose Lo, Yu Rong Mao, Le Li, Ai Hua Lin, Jiang Li Zhao, Ling Chen, Qiang Lin, Hai Li, Dong Feng Huang

**Affiliations:** 1 Department of Rehabilitation Medicine, Guangdong Engineering and Technology Research Centre for Rehabilitation Medicine and Translation, The First Affiliated Hospital, Sun Yat-sen University, Guangzhou, 510080 China; 2 Faculty of Medical Statistics and Epidemiology, School of Public Health, Sun Yat-sen University, Guangzhou, 510080 China

## Abstract

**Background:**

Multisensory interactive training has an increasingly prominent role in stroke rehabilitation. Currently, there is insufficient evidence to demonstrate its efficacy on gait improvement, upper limb and lower limb functional improvement, global motor function and cognitive improvement. A recent Cochrane review confirmed that published studies on virtual reality (VR) training have the limitations of lack of powered sample size, did not evaluate the benefits over a long-term period and lacked trial quality on cognitive function. Another systematic review also concluded that the evidence for the use of VR in gait and balance improvement is limited. This study investigates the effects of multisensory training on gait pattern, upper and lower limb biomechanics, upper limb gross and fine motor functions, and lower limb functional recovery over a medium- to long-term period.

**Methods:**

Two hundred and twenty-four acute stroke patients will be recruited from a single centre over a period of 6 years. Participants will be randomly assigned to either conventional therapy or conventional therapy with VR training. Outcomes will be recorded at baseline, post intervention and at 3, 6 and 12 months post intervention. Primary outcome measure is gait speed. Secondary outcome measures include kinematic data of upper and lower limb motion, muscle tone, Action Research Arm Test and Short Orientation Memory Concentration Test.

**Discussion:**

The results of this trial will provide in-depth understanding of the effect of early VR interventions on gait, upper and lower limb biomechanics and how it may relate to changes in functional outcomes and muscle tone.

**Trial registration:**

Chinese Clinical Trial Registry (Registration No.: ChiCTR-IOC-15006064). Registered on 11 May 2015.

**Electronic supplementary material:**

The online version of this article (doi:10.1186/s13063-017-1874-y) contains supplementary material, which is available to authorized users.

## Background

Stroke is the third leading cause of disability worldwide [1, 2]. Stroke survivors often have an abnormal hemiparetic gait pattern that is characterised by decreased gait speed [3], altered kinematics of hip, knee and ankle joints during a gait cycle [4] and functional impairment of lower and upper extremities. Impaired ability to walk is one of the most devastating consequences and gait recovery is often regarded as a primary objective in stroke rehabilitation [5]. It is becoming widely recognised that multisensory VR training is an important component of stroke rehabilitation and one that has demonstrated potential to promote gait, lower and upper limb functional recovery, and thus increase quality of life.

Virtual reality (VR) has been subjected to intense research over the past decade as an intervention to promote functional recovery for patients with stroke. The virtual environment offers multisensory interaction and goal-orientated tasks that can stimulate active repetitive movements and offer instant feedback [6]. VR is relatively low cost and may be employed within home settings where patients live remotely or have other barriers to participate in intervention [7]. The concept of using commercial gaming (CG) systems to deliver VR interventions at home as part of the rehabilitation programme was well received among stroke survivors as they believed it to be beneficial for functional recovery [8]. Previous studies on VR interventions indicated that they may be more effective in improving gait, lower limb functions [9, 10] and upper limb functions [11] when compared to conventional therapy. Functional imaging studies have indicated that cortical reorganisation is associated with gait improvement post-VR interventions. The latest published meta-analysis [7] suggested that VR interventions are at least as effective as conventional physiotherapy in improving lower limb outcomes within the stroke population.

Gait performance is a predictor for disability [12, 13], mortality and morbidity [14]. Early intervention with physical therapy to restore gait function had been shown to improve motor function and decrease the subsequent disability when compared to intervention provided at the subacute or chronic stages [15]. Despite the reported positive results for VR interventions, published systematic reviews concluded that the evidence for VR in gait, lower limb functions and balance improvement is limited [16], particularly for stroke patients during the acute stage [17]. It was argued that since most of the neuroplasticity activity took place within the first month of stroke occurrence, VR intervention provided during the acute stage of stroke is likely to lead to better clinical outcomes for gait and lower limb functions [18]. A Cochrane review [19] also concluded that there was insufficient evidence to demonstrate the efficacy of VR training on gait speed and global motor function improvement due to a lack of powered sample size and did not evaluate the effectiveness of VR over a long-term period or post hospital discharge. Thus, little is currently known about the impact of VR on gait and lower limb functions during the acute stage and if the impact is sustainable in the long term.

The recovery of upper limb function to perform delicate motions, such as grasping, finger pinching and individual finger movement continues to be a challenge for stroke survivors [11]. Existing rehabilitation programmes, such as constrain-induced therapy, imagery training and bilateral training, have focussed on the improvement of upper limb gross motor skills [20–22]. These training regimes have insufficient emphasis on fine motor skills and coordination of limb movements; thus, leading to reduced ability to carry out activities of daily living. Sophisticated robotic devices have been developed to provide the possibility to train hand and finger motor skills [11]. However, these types of devices are not easily accessible in most rehabilitation departments. In addition, fine motor movement involves the combination of cognitive and motor functions to plan and execute it. Cognitive function refers to the ability to process received information, and to take appropriate action accordingly [12]. Large numbers of stroke survivors have cognitive impairment which presents an additional challenge in upper limb functional recovery. Previous studies have indicated that cognitive function can be recovered through repetitious training [13]. Two Cochrane reviews [19, 23] suggest that VR interventions appear to be promising with moderate-quality evidence to support the beneficial effects on upper limb functional improvement. However, the number of studies available are too few and too small to draw conclusions on upper limb recovery and cognitive function improvements induced by VR interventions. In addition, most of the studies assessed gross motor function by functional outcome measures such as the Action Research Arm Test and the Fugl-Meyer Assessment of Motor Recovery. While they are validated clinical tests, several authors cast doubts on the use of functional outcomes to study motor recovery post stroke. It was argued that functional outcome measures only focussed on task accomplishment regardless of the quality of movement [24, 25]. Since motor compensation can affect [26, 27] task accomplishment, it is, therefore, essential to understand the impact of VR on motor performance measures and to cross reference with functional outcome measures.

## Hypotheses

The primary aim of this study is to test the hypotheses that patients with stroke who receive VR training during the acute stage will have significantly higher improvement in gait speed, lower limb motor functions and upper limb gross and fine motor functions when compared to those who receive conventional therapy. The observed improvements in the VR group will remain significantly higher than the conventional therapy group at 3-, 6- and 12-month follow-up periods. The secondary aim of this study is to assess the changes of the kinematic characteristics of gait and upper limb motions of ‘reach forward’ and ‘reach forward and grasp’ after 3 weeks of VR interventions and at 3-, 6- and 12-month follow-up periods.

## Methods

### Study design

This study is a single-blind randomised controlled trial (RCT) to compare the differences between two parallel groups.

### Study setting

This is a single-centre study which will be conducted by the Rehabilitation Department of the Sun Yat-sen University, China. Participants will be recruited from the inpatient ward. All interventions will be delivered within the Hospital Rehabilitation Department by hospital staff.

### Recruitment

Patients who are admitted to the inpatient ward will be screened for eligibility as part of routine assessment. Suitable participants will be identified by the clinical team and given written information about the study. They will then be approached by a member of the research team to inquire whether they are interested and willing to take part in the study. Written consent will be obtained from participants who are willing to participate. A screening log of all nonrecruited patients and reasons for exclusion will be maintained.

### Randomisation

Participants will be randomly allocated to the control or VR groups. A randomisation schedule will be pre-generated by the permuted block randomisation technique, using blocks of 10 participants with a 1:1 allocation ratio. The randomisation schedule will be calculated in SPSS by a statistical expert from the Faculty of Medical Statistics and Epidemiology, Sun Yat-sen University. The sequences of allocation are kept in a sealed envelope. The randomisation process will allocate each participant an identification number, which will appear on all report forms to maintain confidentiality.

### Sample population

This study has the following inclusion criteria: (1) within 1 month of the first occurrence of stroke, (2) stage 2 of the Brunnstrom classification, (3) magnetic resonance imaging (MRI)- or computed tomography (CT)-confirmed stroke, (4) age between 40 and 80 years, (5) have at least 20° of wrist flexion/extension and at least 10° of finger flexion and extension of the affected limbs, (6) be able to walk at least 10 m with or without assistance and (7) no severe cognitive impairment (Mini Mental State Examination score below 10 [28]).

This study will exclude participants who are: (1) medically unstable, (2) have already received elements of the training programme over 1 week and (3) have brain stem injury.

## Ethics

The study was approved by the Medical Ethical Committee of the First Affiliated Hospital of Sun Yat-sen University (Ethics no.: [2014] 88). The clinical trial is registered with the Chinese Clinical Trial Registry (Registration No.: ChiCTR-IOC-15006064, registered on 11 March 2015). Any important protocol modifications will be communicated with all relevant parties. All patients who meet the criteria will be invited to take part in the study. Patients will be given time to consider whether they wish to take part in the trial and to ask any questions. All participants can withdraw from the trial at any time without giving reason. Participants can decide if they wish to withdraw completely, in which case all collected data will be excluded from the study, or to withdraw only from further assessment or intervention wherein all collected data will be included. All participants will be reimbursed for travel expenses to encourage adherence during the follow-up phase.

## Outcome measures

### Primary outcome measure

Gait speed, assessed by the 10-m Walk Test, is the primary outcome measure.

### Secondary outcome measures

**Fig. 1:**
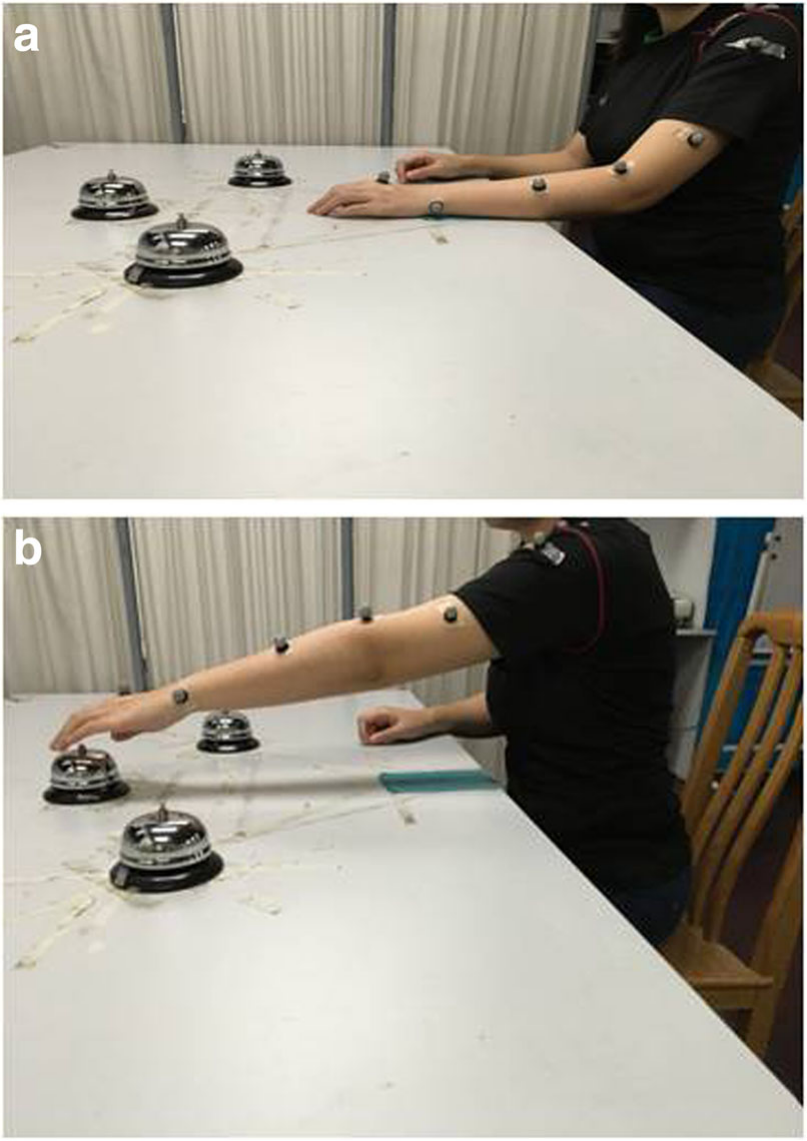
**a** Starting position of reach forward assessed by Vicon. **b** End position of reach forward motion

**Fig. 2:**
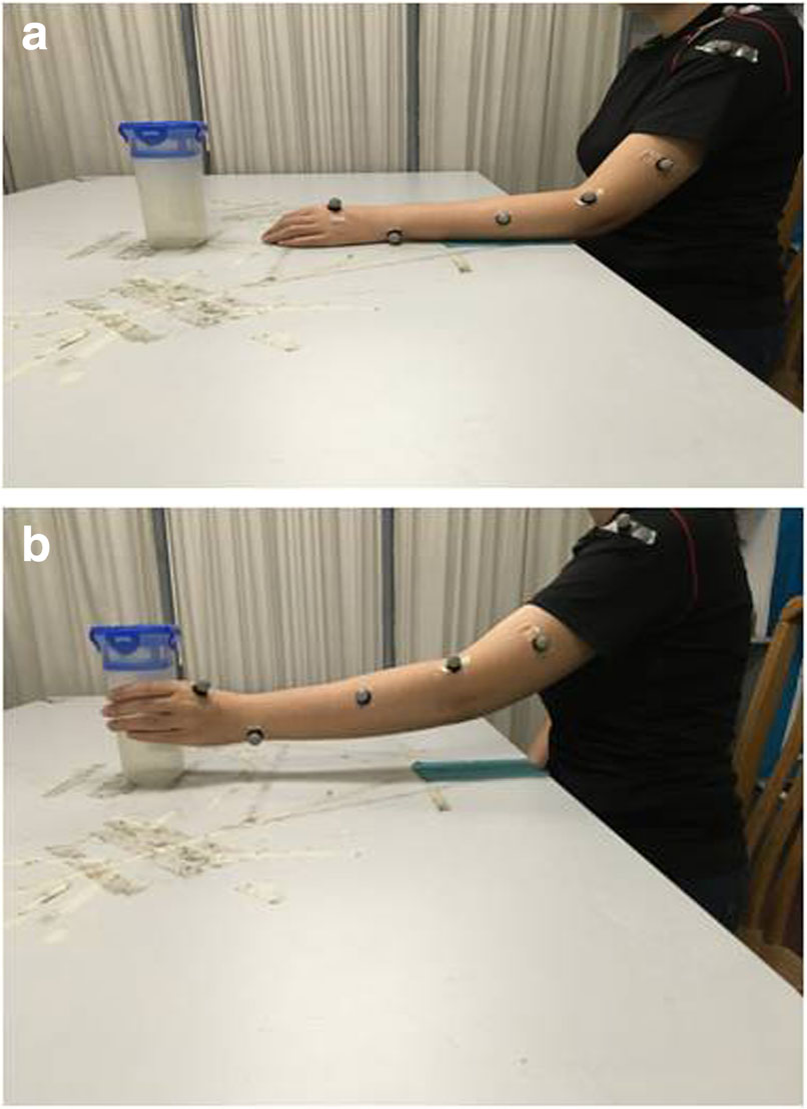
**a** Starting position of reach and grasp motion assessed by Vicon. **b** End position of each and grasp motion

Secondary outcome measures include 3D motion analysis of upper limb reach forward and reach forward and grasp motions (Figs. 1 and 2) and gait. A breif description of each secondary outcome measure is presented in Table 1. Outcome measures will be recorded at baseline, post intervention and at 3, 6 and 12 months post intervention.

**Table 1:** Descriptions of the secondary outcome measures

Secondary outcome measures	Descriptions
Action Research Arm Test (ARAT)	The ARAT [41] is a 19-item observational test. It is divided into 4 subtests (grasp, grip, pinch and gross arm movement). Performance on each item is rated on a 4-point ordinal scale ranging from: 0 – can perform no part of test; 1 – performs test partially; 2 – completes test, but takes abnormally long or has great difficulty; 3 – performs test normally
Instrumental Activities of Daily Living (IADL)	The IADL [24] is a functional disability scale which assesses functional level by asking whether a person receives personal help with activities of daily living, such as using the telephone, getting to places outside the house, grocery shopping, preparing meals, doing housework or handyman work, laundry, taking medications and managing finances
Gait analysis	Spatiotemporal gait parameters, joint angles and moment of the lower limbs will be recorded by Vicon Motion Systems, Oxford, UK. Parameters will be recorded at: (1) maximum hip and knee extension during stance phase, (2) maximum flexion at the hip and knee joints during the swing phase, (3) plantarflexion during push-off and (4) dorsiflexion during swing phase of the gait cycle. Spatiotemporal gait parameters of gait speed, cadence, stride length, stride time and step length will be recorded
Upper limb motion analysis	Dynamic changes of shoulder and elbow motions will be measured by Vicon Motion Systems. Parameters recorded during reach forward task (Fig. 1a, b) are: (1) movement time (s), (2) peak velocity (m/s), (3) shoulder amplitude (^o^) and (4) elbow amplitude (^o^)
	Parameters of: (1) movement time (s), (2) peak velocity (m/s), (3) shoulder amplitude (^o^) and (4) elbow amplitude (^o^), (5) supination peak velocity (m/s) and (6) pronation peak velocity (m/s) during reach forward and grasp (Fig. 2a, b)
	Motions for the paretic and nonparetic arms will be recorded and compared
Short Orientation Memory Concentration Test (SOMCT)	The SOMCT [25] is a validated measurement for cognitive impairment. It is a short assessment of cognitive function composed of 6 items
Mini Mental Status Examination (MMSE)	The MMSE is a brief screening tool that provides a quantitative assessment of cognitive impairment and assesses changes over time. It was originally developed as part of an assessment for dementia and was validated for use in patients with acute stroke [26]
Fugl-Meyer Assessment of Motor Recovery (FMA)	The FMA [27] is a validated evaluation tool for motor function, balance and joint function in post-stroke hemiplegic patients
Berg Balance Scale (BBS)	The BBS [42] was developed to assess static and dynamic balance abilities. It is composed of 14 simple balance-related tasks including sit to stand, stand to sit and standing on one foot
Grip strength	Grip strength will be measured with a Jamar ® hydraulic hand dynamometer
Muscle tone	Changes of muscle tone (bicep brachii, brachioradialis, extensor digitorum, flexor carpi radialis and flexor carpi ulnaris) pre and post treatment will be measured by the MyotonPRO® hand-held device whose use in the stoke population has been validated [43]
World Health Organisation Disability Assessment Schedule 2.0 (WHODAS)	The WHODAS is a standardised measurement of health and disability across cultures [44]. It captures the level of functioning in 6 domains of life: cognition, mobility, self-care, getting along, life activities and participation. It has been validated for use in people with chronic illnesses [45]

### Outcome assessments

Outcome assessments will be conducted by the dedicated assessment team. The team consists of occupational therapists, physiotherapists, physicians and engineers who are specifically trained to perform the outcome assessments. The institute is a World Health Organisation Collaborating Centre and is responsible for delivering training on the use of the outcome measure tools to health care professionals.

## Sample size

This study uses gait speed as the primary outcome measure for sample size calculation. An improvement in gait speed of 0.16 m/s was recommended as the minimum clinically significant difference during the first 60 days post stroke [29]. The sample size was determined by a statistical expert, using the formula:

$$ N=\frac{{\left({Z}_{\alpha}+{Z}_{\beta}\right)}^2{\sigma}^2}{\delta^2}\left({Q_1}^{-1}+{Q_2}^{-1}\right) $$

where *Z*_*α*_ is normal distribution quantiles for type I error, *Z*_*β*_ is normal distribution quantiles for type II error, σ is pooled standard deviation derived from past gait speed data, *δ* is difference in gait speed between the intervention and control group, and *Q* and *Q* are the proportions of control and intervention group.

Based on a 10% missing rate, each group requires 112 (total 224) participants to give 90% power to detect a gait speed difference of over 0.3 m/s.

## Procedure

**Fig. 3:**
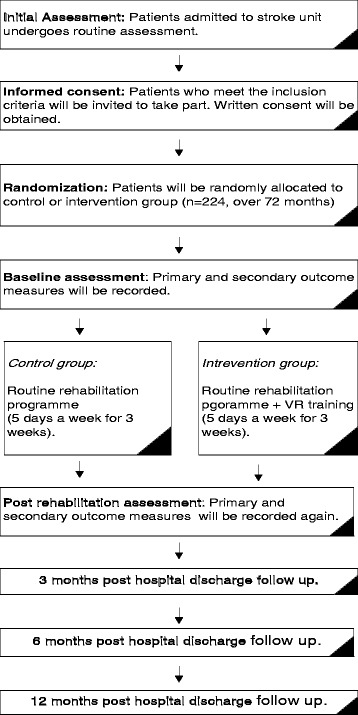
Flow diagram for the study procedure

**Fig. 4:**
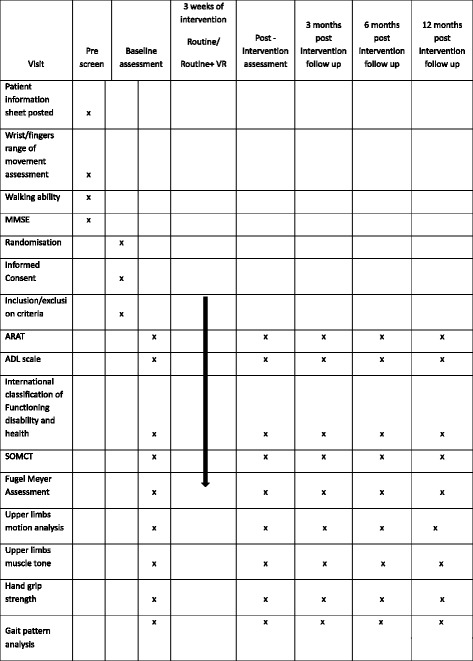
Standard Protocol Items: Recommendations for Interventional Trials (SPIRIT) figure. Overview of the schedule of events

The study procedure is illustrated in Fig. 3. The Standard Protocol Items: Recommendations for Interventional Trials (SPIRIT) figure shows the overview of the schedule of events (Fig. 4) (Additional file 1).

### Control group

Participants in the control group will receive routine medical care and participate in conventional physiotherapy and occupational therapy. Therapeutic programmes include lower limb strengthening exercises and traditional gait and balance training, upper limb functional training and activities of daily living practice. The rehabilitation programme will be provided for 5 days a week for 3 weeks, with each therapy session lasting for 1 h (total of 2 h).

### Intervention group

The intervention group will participate in a multisensory VR training programme for 5 days a week for 3 weeks. VR training is provided in addition to the routine programme. It consists of four components: gait training, limb coordination, gross motor function and fine motor function.

### Gait training

**Fig. 5:**
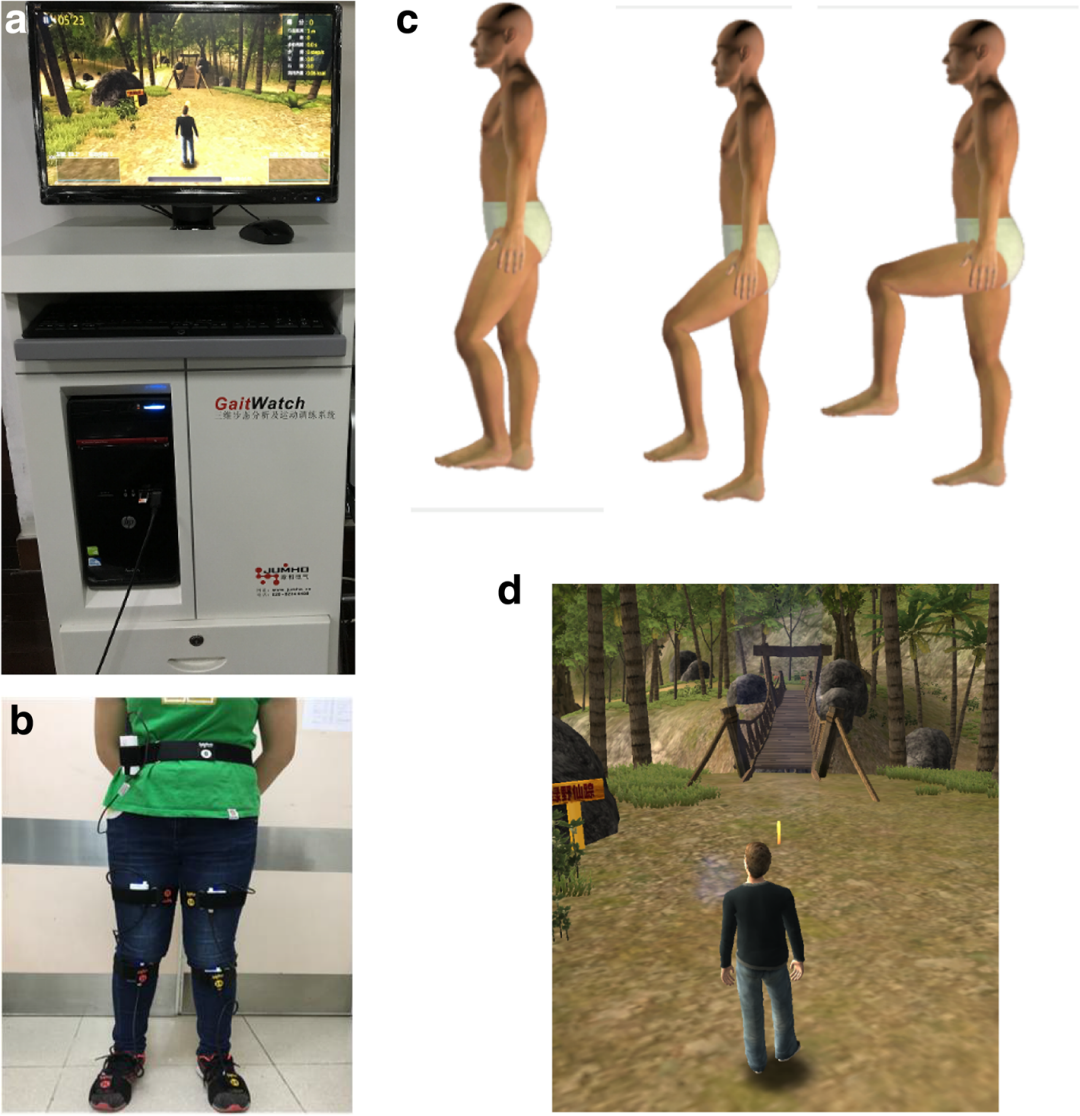
GaitWatch system. **a** GaitWatch hardware. **b** Sensor position. **c** Real-time feedback displayed on the screen. **d** Walking tasks participants will be asked to complete

Gait training will be provided through the use of the GaitWatch system (JumHo, China). The system requires the use of seven transmitters strapped to the following points: the lumbar spine at the L4/L5 level, the mid-portion of the femur, the tibial tuberosity and the mid-foot region (on both legs). Figure 4 shows the GaitWatch system and the locations of the transmitters (Fig. 5a, b). GaitWatch provides a gait training programme with bespoke gaming activities, which include marching on the spot, lifting hips and knees to required position or walking in a virtual environment (Fig. 5c, d). Real-time feedback on the quality of movement will be provided to the participants. Figure 4 shows the GaitWatch system and the locations of the transmitters. Training sessions will last up to 20 min (excluding a 5-min break at the halfway mark). The system will produce a report on gait speed, maximum joint angle achieved, weight distribution and percentage of deviation from expected motion trajectory.

### Limb coordination and gross upper limb motor function

**Fig. 6:**
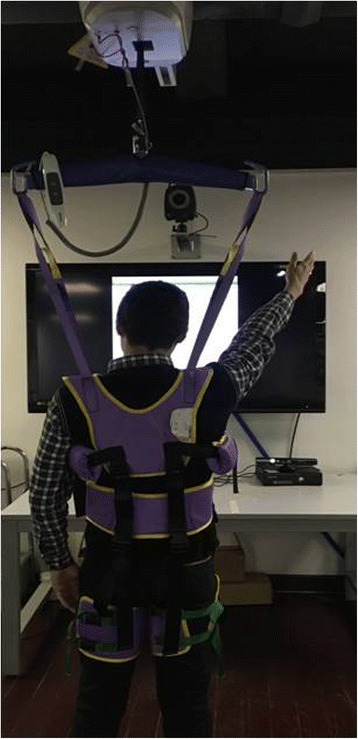
A body-weight support harness will be provided to those who are not able to stand for a sufficient length of time

Limb coordination and gross motor function will be trained through use of the Microsoft Xbox 360 Kinect via the games Adventures and Fruit Ninja. Participants will be asked to perform tasks that are displayed on the screen at progressive difficulties. The Adventures game will encourage balance, mobility and stepping. Fruit Ninja will encourage shoulder flexion/extension and internal/external rotation and elbow flexion and extension. Body motion is captured by the associated motion-capturing device. A body-weight support harness will be provided to those who are not able to stand for a sufficient length of time (Fig. 6). The score achieved during the virtual game will be recorded to monitor progress. Each training programme will last for 10 min (total of 20 min training time in addition to a 5-min break), 5 days a week for 3 weeks.

### Fine motor function

**Fig. 7:**
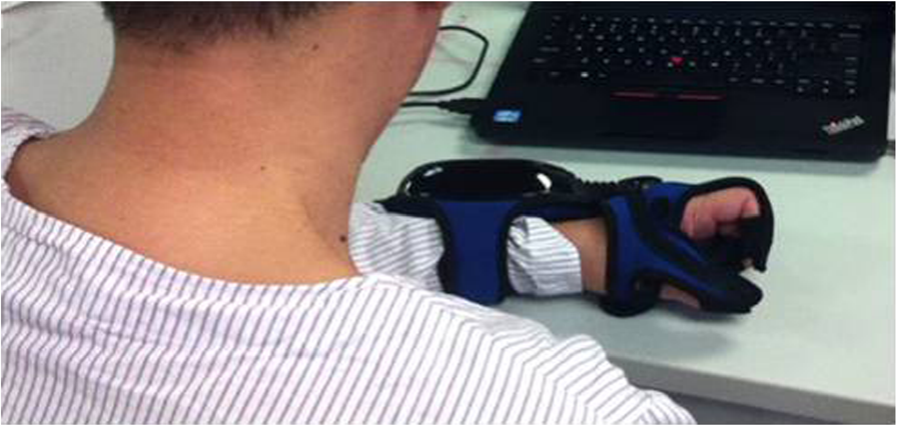
HandTutor® glove for fine motor training

The fine motor function of the upper limbs will be trained using the HandTutor™ glove (Fig. 7) with MediTutor software (Meditouch Ltd., Rotem Industrial Park, MP Arava, Israel). The device delivers training via bespoke games. The gloves capture the biomechanical motions of the fingers and wrists. Exercises can be tailored to train all fingers or isolated finger movements. Real-time feedback of quality of movement is displayed on the screen. The previously published exercise programme ‘Track’ [30] will be used as the exercise for this study. ‘Track’ is a programme where a ball moves along a track horizontally across the screen at varying heights. The movement of the ball is controlled by flexion and extension of the finger/s or wrist. The participant’s task is to keep the ball within the track by flexing and extending the finger/s or wrist, which changes the upward and downward gradients of the ball. The therapist can set the participant’s range of motion that will be exercised by changing the height of the track. The maximum range of movement represents the full vertical displacement of the ball on the screen. The measurement is a linear measurement and treats the fingers as a single joint. Fine motor training will last for 20 min (with a 5-min break every 6 min), 5 days a week for 3 weeks.

### Safety and adverse event reporting

The participants recruited into this study are patients with acute stroke with a likelihood of morbidity and mortality. Procedures are, therefore, in place to minimise adverse events. The risk relating to the intervention is minimised as patients who are medically unstable will be excluded from the study. Participants will not be asked to exercise beyond their physical ability. The exercise sessions will be delivered by trained physiotherapists/occupational therapists who will work closely with the inpatient clinical team, and they will have regular contact and access to the inpatient clinical team for any queries that may arise. The trial will adhere to the following safety reporting procedures:

1. All serious adverse events (SAEs) related to the interventions will be recorded on an SAEs Event Report Form in accordance with local hospital procedure. A SAE is defined as an event that may harm the participant and may require medical or surgical intervention to prevent one of the following outcomes: death, a life-threatening situation, impairment of a body function, permanent damage to a body structure, and prolonged hospitalisation
2. All SAEs related to the interventions must be reported to the principle investigator within 24 h of learning of the event
3. SAEs that are related to the interventions will be reported to the special committee of the University Clinical Trial Unit and Research Ethics Committee within 15 days of the principle Investigator becoming aware of the event. Information that will be reported to relevant parties is: (1) appropriate identifying information for the research protocol, (2) a detailed description of the SAE and its outcome, (3) a description of any changes to the protocol or other corrective actions that have taken, or are proposed in response to the SAE

## Data management

A bespoke data management system was developed by Rilintech Co., Ltd. (Beijing,China) for data storage. The assessment session and data entry are always conducted with at least two assessors to minimise data entry error. Monitoring of study conduct and data collection will be performed by a special committee of the University Clinical Trial Unit on a biannual basis. Recorded data are only accessible by authorised personnel.

## Data analysis

Descriptive statistics will be used to describe the demographic data of each sample group. The normality of each parameter will be checked by histogram and the Kolmogorov-Smirnov test.

Chi-squared tests will be used to assess sample characteristics of gender and types of cerebral infarction.

The pre- and post-intervention differences in gait speed within individuals and between groups will be assessed by paired-samples *t* tests (two-tailed) and one-way analysis of variance (ANOVA), respectively. The comparison of 3-month, 6-month and 1-year follow-ups will be assessed by repeated-measures ANOVA.

A broadly similar approach will be used to analyse the secondary outcome measures of muscle tone, other kinematic parameters of gait, reach forward motion and reach forward and grasp motion. The kinematic parameters of the paretic side will be compared with the nonparetic side at all recording points using paired-samples *t* tests.

The Mann-Whitney *U* test will be used to test the between-group differences for the ordinal data obtained from the functional outcome measures and WHODAS 2.0.

The differences between the two groups during the follow-up phase will be assessed by the Kaplan-Meier test.

There are no planned interim analyses or stopping rules, as the power calculation has accounted for loss to follow-up. All analyses will initially be performed on an intention-to-treat basis.

## Discussion

The VR intervention works on the principle of neuroplasticity [31]. Studies from the past decade suggest that different types of VR can increase gait speed, lower and upper limb functions and kinematic parameters. There are a number of clinical guidelines which recommend active rehabilitation to be commenced within 24 to 48 h of stroke occurrence [32, 33] as the greatest part of the recovery was reported to take place in the first month following stroke [34]. There is some evidence to demonstrate the benefit of an early exercise programme on functional recovery but evidence for early VR intervention is lacking. The majority of the published studies focussed on patients with subacute and chronic stroke [16, 23]. Thus, this study will provide evidence for the use of VR as part of an early intervention programme.

The present study also assesses the medium- to long-term impact of VR interventions for patients with stroke during the acute stage. The Cochrane reviews published in 2011 and 2015 [6, 19] both concluded that it was unknown whether the effects of VR were sustained in the longer term. The review [35] found that only one study [36] out of the 37 included studies had measured the effects of VR on upper limb functions at the 6-month follow-up. Motor capacities evolve most strongly over the first month post stroke [37] and intervention provided at an early stage has better clinical outcomes when compared to intervention provided at a later stage of stroke. Thus, the follow-up data record at 3, 6 and 12 months post intervention will increase the understanding of whether early VR intervention is superior to conventional therapy in the longer term.

The kinematic data of gait and upper limb motion will help to establish if functional improvement induced by early VR intervention is associated with changes of kinematic patterns. Buma [38] proposed that the desired outcome of stroke rehabilitation was to promote task completion in a manner that is close to normal movement pattern. Detailed kinematic information would enable the identification of potential compensation movement. Nonbiomechanical outcome measures had in the past misled clinicians to believe that a faster observed speed of reach forward movement execution may improve movement quality as appreciated by the smoothness [39]. However, recent biomechanical study has revealed that the faster execution speed observed in patients with stroke was related to trunk compensation movement [40]. A kinematic study is, therefore, essential to assess whether VR intervention is effective in promoting normal movement patterns as well as functional improvement.

In conclusion, this current research is set to provide information to demonstrate the clinical effectiveness of VR as part of an early intervention programme for patients with stroke.

## Trial status

Recruiting since May 2015.

## Additional file

Additional file 1: SPIRIT 2013 Checklist: recommended items to address in a clinical trial protocol and related documents*. (DOCX 50 kb)

## Abbreviations

ARAT: Action Research Arm Test
CG: Commercial gaming
CT: Computed tomography
MMSE: Mini Mental State Examination
MRI: Magnetic resonance imaging
VR: Virtual reality

## References

[1] Strong K, Mathers C, Bonita R (2007). Preventing stroke: saving lives around the world. Lancet Neurol.

[2] Langlois JA, Rutland-Brown W, Wald MM (2006). The epidemiology and impact of traumatic brain injury: a brief overview. J Head Trauma Rehabil.

[3] Olney SJ, Richards C (1996). Hemiparetic gait following stroke. Part I: Characteristics. Gait posture..

[4] Mao YR (2015). The effect of body weight support treadmill training on gait recovery, proximal lower limb motor pattern, and balance in patients with subacute stroke. Biomed Res Int..

[5] Yang YR (2008). Virtual reality-based training improves community ambulation in individuals with stroke: a randomized controlled trial. Gait Posture.

[6] Laver KE (2011). Virtual reality for stroke rehabilitation. Cochrane Database Syst Rev..

[7] Gibbons EM, Thomson AN, De NM (2016). Are virtual reality technologies effective in improving lower limb outcomes for patients following stroke - a systematic review with meta-analysis[J]. Top Stroke Rehabil.

[8] Paquin K, Crawley J, Harris JE (2016). Survivors of chronic stroke – participant evaluations of commercial gaming for rehabilitation[J]. Disabil Rehabil.

[9] You SH (2005). Virtual reality-induced cortical reorganization and associated locomotor recovery in chronic stroke: an experimenter-blind randomized study. Stroke.

[10] Kim JH (2009). Use of virtual reality to enhance balance and ambulation in chronic stroke: a double-blind, randomized controlled study. Am J Phys Med Rehabil.

[11] Ockenfeld C (2013). Fine finger motor skill training with exoskeleton robotic hand in chronic stroke: stroke rehabilitation. IEEE Int Conf Rehabil Robot..

[12] Bohannon RW, Andrews AW, Glenney SS (2013). Minimal clinically important difference for comfortable speed as a measure of gait performance in patients undergoing inpatient rehabilitation after stroke. J Phys Ther Sci.

[13] Collen FM, Wade DT, Bradshaw CM (1990). Mobility after stroke: reliability of measures of impairment and disability. Int Disabil Stud.

[14] Jiang B (2006). Incidence and trends of stroke and its subtypes in China: results from three large cities. Stroke.

[15] Hesse SA (1994). Gait outcome in ambulatory hemiparetic patients after a 4-week comprehensive rehabilitation program and prognostic factors. Stroke.

[16] Luque-Moreno C (2015). A decade of progress using virtual reality for poststroke lower extremity rehabilitation: systematic review of the intervention methods. Biomed Res Int..

[17] Chen L, et al. Effect of virtual reality on postural and balance control in patients with stroke: a systematic literature review. Biomed Res Int. 2016;2016(2016):Article ID 7309272. 10.1155/2016/7309272 PMC5174165 28053988

[18] Langhorne P, Bernhardt J, Kwakkel G (2011). Stroke rehabilitation. Lancet.

[19] Laver KE (2015). Virtual reality for stroke rehabilitation. Cochrane Database Syst Rev..

[20] Taub E (2013). Method for enhancing real-world use of a more affected arm in chronic stroke: transfer package of constraint-induced movement therapy. Stroke.

[21] Cho HY, Kim JS, Lee GC (2013). Effects of motor imagery training on balance and gait abilities in post-stroke patients: a randomized controlled trial. Clin Rehabil.

[22] Sleimen-Malkoun R (2011). Bimanual training in stroke: how do coupling and symmetry-breaking matter?. BMC Neurol..

[23] Pollock A (2014). Interventions for improving upper limb function after stroke. Cochrane Database Syst Rev..

[24] Lawton MP, Brody EM (1969). Assessment of older people: self-maintaining and instrumental activities of daily living. Gerontologist.

[25] Katzman R (1983). Validation of a short Orientation-Memory-Concentration Test of cognitive impairment. Am J Psychiatry.

[26] Nys GM (2005). Restrictions of the Mini-Mental State Examination in acute stroke. Arch Clin Neuropsychol.

[27] Fugl-Meyer AR (1975). The post-stroke hemiplegic patient. 1. A method for evaluation of physical performance. Scand J Rehabil Med.

[28] Folstein MF, Folstein SE, Mchugh PR (1975). ‘Mini-mental state’. A practical method for grading the cognitive state of patients for the clinician. J Psychiatr Res.

[29] Tilson JK (2010). Meaningful gait speed improvement during the first 60 days poststroke: minimal clinically important difference. Phys Ther.

[30] Carmeli E (2011). HandTutor enhanced hand rehabilitation after stroke—a pilot study. Physiother Res Int.

[31] Fluet GG, Patel J, Qiu Q, Yarossi M, Massood S, Adamovich SV, Tunik E, Merians AS. Motor skill changes and neurophysiologic adaptation to recovery-oriented virtual rehabilitation of hand function in a person with subacute stroke: a case study. Disabil Rehabil. 2016. doi:10.1080/09638288.2016.1226421. 10.1080/09638288.2016.1226421 PMC5368038 27669997

[32] National Stroke Foundation. Clinical Guidelines for Stroke Management 2010. Melbourne; 2010. p. 1–167.

[33] Indredavik B (1991). Benefit of a stroke unit: a randomized controlled trial. Stroke.

[34] Wade DT (1983). The hemiplegic arm after stroke: measurement and recovery. J Neurol Neurosurg Psychiatry.

[35] Laver K (2015). Virtual reality for stroke rehabilitation: an abridged version of a Cochrane review. Eur J Phys Rehabil Med.

[36] Housman SJ, Scott KM, Reinkensmeyer DJ (2009). A randomized controlled trial of gravity-supported, computer-enhanced arm exercise for individuals with severe hemiparesis. Neurorehabil Neural Repair.

[37] van Dokkum L (2014). The contribution of kinematics in the assessment of upper limb motor recovery early after stroke. Neurorehabil Neural Repair.

[38] Buma F, Kwakkel G, Ramsey N (2013). Understanding upper limb recovery after stroke. Restor Neurol Neurosci.

[39] Dejong SL, Schaefer SY, Lang CE (2012). Need for speed: better movement quality during faster task performance after stroke. Neurorehabil Neural Repair.

[40] Mandon L (2016). Faster reaching in chronic spastic stroke patients comes at the expense of arm-trunk coordination. Neurorehabil Neural Repair.

[41] Lyle RC (1981). A performance test for assessment of upper limb function in physical rehabilitation treatment and research. Int J Rehabil Res.

[42] Berg KO (1992). Measuring balance in the elderly: validation of an instrument. Can J Public Health..

[43] Frohlich-Zwahlen AK (2014). Validity of resting myotonometric assessment of lower extremity muscles in chronic stroke patients with limited hypertonia: a preliminary study. J Electromyogr Kinesiol.

[44] Ustun T, et al. Measuring health and disability. Manual for WHO Disability Assessment Schedule WHODAS 2.0. Geneva: World Health Organisation; 2010.

[45] Cheung MK (2015). Validation of the World Health Organization Assessment Schedule II Chinese Traditional Version (WHODAS II CT) in persons with disabilities and chronic illnesses for Chinese population. Disabil Rehabil.

